# Preparation and Evaluation of Imatinib Loaded Transfersomal gel for the Treatment of Rheumatoid Arthritis

**DOI:** 10.22037/ijpr.2021.115481.15394

**Published:** 2021

**Authors:** Somayeh Taymouri, Valiollah Hajhashemi, Majid Tabbakhian, Massoud Torkashvand

**Affiliations:** a *Department of Pharmaceutics, School of Pharmacy and Novel Drug Delivery Systems Research Centre, Isfahan University of Medical Sciences, Isfahan, Iran.*; b *Department of Pharmacology, School of Pharmacy, Isfahan University of Medical Sciences, Isfahan, Iran.*

**Keywords:** Imatinib, Rheumatoid arthritis, Transfersomes, D-optimal design, skin permeation studies

## Abstract

In the present study, imatinib-loaded transfersomal gel (imatinib-TFS-Gel) was developed to minimize the oral dosing frequency and side effects during rheumatoid arthritis (RA) therapy. Imatinib-loaded transfersomes (imatinib-TFS) were prepared by the film-hydration method. The effects of lecithin content, lecithin/ EA ratio, and the type of EA on the characteristics of the imatinib-TFS were studied using a D-optimal design. Morphology of imatinib-TFS was investigated using scanning electron microscopy. The optimized imatinib-TFS formulation was used to prepare imatinib-TFS-Gel with the aid of Carbopol 940 as the gelling agent. The Optimized imatinib-TFS had a spherical shape with the particle size of 140.53 ± 0.87 nm, polydispersity index of 0.44 ± 0.01, the zeta potential of -17.63 ± 0.65 mV, encapsulation efficiency of 98.70 ± 0.38%, and release efficiency of 81.26 ± 0.70 %. *Ex-vivo* skin permeation studies through the rat skin showed that the cumulative amount of imatinib permeated from imatinib-TFS-Gel was significantly higher than that from imatinib-Gel. The RA rat model indicated a substantial reduction in paw edema during the 14 days study following the application of imatinib-TFS-Gel as compared with imatinib-Gel. Therefore, imatinib-TFS-Gel can be considered as a promising drug delivery system for the treatment of RA.

## Introduction

Rheumatoid arthritis (RA) is a progressive and chronic inflammatory disease in joints affecting nearly 1% of the world population. It is caused by the invasion of immune cells into the synovium, followed by proliferation of synovial lining cells and development of pannus (1). According to reports in the literature, women are more affected than men because of hormonal impacts on immune cells. The etiology of RA is still not completely known, however, a complex interaction between numerous genetic and environmental factors may be involved. RA is associated with synovial hyperplasia, pannus formation and destruction of cartilage articular bone. Its clinical symptoms are mainly joint stiffness, pain, swelling, deformity, tenderness and redness in affected joints (2). As a selective inhibitor of a number of tyrosine kinases, imatinib has shown high efficiency in the treatment of RA. It acts via occupying the ATP binding site in the tyrosine kinase domain of the c-KIT receptor and platelet-derived growth factor receptor (PDGF-R) and macrophage c-fms receptor. Mast cells are potent immune cells that are frequently found in arthritic joints and imatinib can induce selective apoptosis in synovial mast cells by inhibiting the KIT tyrosine kinase signaling pathway. As a result, the production of potent pro-inflammatory mediators such as Tumor necrosis factor (TNF-α) and interleukin (IL)-6 is decreased (3). Imatinib may also inhibit the PDGF-stimulated proliferation of synovial fibroblasts and c-fms-mediated macrophage maturation and thereby reduce the production of TNF-α (4-5). Imatinib, when administered orally, lacks the specific targeting ability resulting in decreased drug distribution to the site of action. As a result, frequent administration of the drug will be required to achieve the drug concentration required at the site of action. This will lead to several adverse reactions including edema, nausea, muscle cramps, musculoskeletal pain, bone marrow suppression, neutropenia, thrombocytopenia, anemia, cardiotoxicity (5). Thus, a topical formulation of imatinib with a high degree of permeation could be advantageous for the treatment of RA. Dermal delivery has several advantages including ease of application, good patient compliance, decreased systemic exposure, and minimized side effects for treating chronic disease (6). The main barrier in percutaneous delivery is inefficient permeation through the stratum corneum (the outermost layer of skin). To overcome this shortcoming, various approaches have been employed, which include iontophoresis, electroporation, phonophoresis, microneedle, chemical methods such as absorption enhancer and colloidal delivery systems. Recently, strategies using liposomal-based delivery systems, such as conventional liposomes, niosomes, transfersomes (TFS), and ethosomes also have been developed and investigated to improve drug permeation through the skin (7). Several studies have shown improved penetration of a variety of drugs incorporated in TFS into deep tissues and to the systemic circulation (8-10). TFS, first discovered by the Cevc group in 1990, are ultra-flexible liposomes that can be made from phospholipids and edge activators (EA). These nanocarriers based systems are composed of at least one aqueous core surrounded by a lipid bilayer. An EA is a single chain surfactant which is used to destabilize the lipid bilayer and render the vesicles ultra-flexibility. Upon application, TFS is able to squeeze themselves to one-tenth of their diameters, and thus, passing as intact vesicles through the intercellular region into the deeper layers under the skin (11-12). TFS has inherently low viscosity, and thus low retention at the application site. Therefore, they are often incorporated in a gel system to increase the formulation contact time and improve drug permeation through the skin. In this study, various formulations of imatinib-loaded TFS (imatinib-TFS) were prepared using the thin-film hydration method. Imatinib-TFS-based gel system was then prepared by the inclusion of Carbopol 940 as a gelling agent and assessed for the drug *ex-vivo* permeation rate, and anti-arthritic effect, as compared with imatinib-Gel.

## Experimental

*Materials*

Imatinib base was kindly gifted by Parsian Pharmaceutical Co. (Iran). Soybean lecithin and the dialysis bag (molecular cut off 12000 Da) were purchased from Sigma (USA). Carbopol 940, Span80, Tween 80 and Span 20 were obtained from Merck (Germany). All other used chemicals were of analytical reagent grade.

*Preparation of imatinib -TFS*

Imatinib-TFS were prepared by the thin-film hydration method. In brief, 5 mg of imatinib, different amounts of lecithin (25-50 mg) and Tween80, Span 80 or Span 20 as EA at the ratios of 4:1, 7:1 and 10:1 were dissolved in 5 mL chloroform. Solvent removal under vacuum using a rotary evaporator (Heidolph, Germany) produced a thin film. The film was hydrated with 5 mL deionized water while rotating at 180 rpm for 30 min at 60 ^o^C. The dispersions were sonicated at 40 W using a probe sonicator (bandelin electronic, Germany) at pulsed on-time 2 sec, off-time 2 sec for 2 min. 

*Experimental* *design *

To assess the effects of lecithin content, lecithin/ EA ratios, and the type of EA on the vesicles properties, a 3-factor, and 3-level D-optimal design were used. Experimental factors and factor levels were chosen based on the results from preliminary studies (Table 1). Design Expert^®^ Software (version 11, Minneapolis, USA) was utilized to generate the design and analyze the results, further (Table 2). The studied responses were particle size (PS), polydispersity index (PDI), zeta potential (ZP), encapsulation efficiency (EE)%, and release efficiency% during 24 h (RE_24 h_%) as described in Table 1. 

*Physicochemical characterization of imatinib-TFS*

*PS, PDI and ZP analysis*

The PS, PDI and ZP of imatinib-TFS were measured by a Zetasizer (PCS, Zetasizer 3000, Malvern, UK). Samples were diluted 5 folds with deionized water before analysis.

*Determination of the EE%*

A volume of 0.5 mL from each formulation was centrifuged (Microcentrifuge Sigma 30 k, UK) at 14000 rpm for 20 min using Amicon® microcentrifugation tubes (cutoff 10000 Da, Ireland). The free (un-encapsulated) imatinib was measured in the filtrate by a UV spectrophotometer (Shimadzu®, Japan) at 260 nm. The EE % was calculated from the following equation:

$\mathrm{EE\%}=(\frac{\mathrm{total amount of drug added}-\mathrm{free drug}}{\mathrm{total amount of drug added}})\times 100$

 Equation 1.

*Drug release studies*

A volume of 0.5 mL from each imatinib-TFS formulation was placed in a dialysis bag, then immersed in 25 mL phosphate buffer solution (PBS, pH 7.4) containing 0.5% Tween 80. The release medium was kept at 37 °C and stirred at 500 rpm. At predetermined time intervals, 0.6 mL samples from the release medium were withdrawn and immediately replaced with fresh PBS. The amount of released drug was determined by UV spectrophotometry at 260 nm, as described previously. To compare the efficiency of drug release among various formulations, RE_24 h_% was determined using the following equation:

${\mathrm{RE}}_{24\mathrm{th}}\%=\frac{\int_{0}^{t}\mathrm{y.dt}}{\mathrm{y100.t}}\times 100$

 Equation 2.

Where y.dt is the area below the release curve up to the time, t, and y100.t reflect the rectangle area defined at the same time by 100% release.

*Scanning electron microscopy (SEM)*

The morphology of the optimized imatinib-TFS was determined using SEM (FEI Co, USA). For this purpose, a drop of the imatinib-TFS was spread onto a carbon-coated copper grid to leave a thin film. The film was coated with gold under vacuum, then examined under SEM.

*Fourier-Transform Infrared (FTIR) Spectroscopy*

The FTIR spectra of imatinib, drug-free TFS, and optimized imatinib–TFS were recorded by FTIR spectroscopy (Rayleigh, WQF-510/ 520, China) using potassium bromide (KBr) pellets technique at room temperature. To form pellets, imatinib, lyophilized drug-free TFS, or optimized imatinib–TFS were thoroughly mixed with KBr. Each KBr pellet was then scanned at the frequency range of 4000–400 cm^−1^.

*Freeze-drying of imatinib–TFS*

The impact of 1% and 3% concentrations of sucrose, mannitol, or lactose as cryoprotective agents on the imatinib-TFS stability throughout freeze-drying was examined. The cryoprotectant was added to the formulation. The samples containing dissolved cryoprotectant were placed in a freezer (set at −20 ^o^C) for 24 h. The frozen samples were immediately subjected to freeze-drying (Christ Alpha2-4 LD plus, Germany) at 0.07 mbar for 48 h. Each lyophilized sample was reconstituted in 2 mL purified water by gently shaking. The reconstitution time, PS, and PDI of samples were measured.

*Preparation of nano-transferosomal gel*

An appropriate amount of Carbopol 940 was added gradually to deionized water under continuous stirring at 500 rpm to form a 0.2% Carbopol dispersion. A known amount of freeze-dried sample from optimized imatinib-TFS formulation was incorporated into Carbopol 940 dispersion to produce a final drug concentration of 0.1% w/v. Finally, the resulting mixture was neutralized with triethanolamine to pH 7 to form Carbopol 940 gel matrix 

*Drug release study from imatinib-TFS-Gel*

Imatinib release from the nano-transferosomal gel was studied using a dialysis bag under the same conditions as discussed in section 2.4.3

*Ex-vivo skin permeation studies*

The rat skin was shaved, excised, and rinsed with physiological saline. Skin samples were clamped between the receptor and donor of Franz diffusion cell with a diffusion area of 4.5 cm^2^. 0.5 mL imatinib-Gel and imatinib-TFS-Gel were put on the surface of the skin in the donor compartment. The donor cell was covered with parafilm to avoid evaporation. The Receiver compartment was filled with 25 mL of PBS (pH 7.4) containing 0.5% Tween 80 and stirred at 500 rpm during the study. The temperature of diffusion cells was kept at 37 ± 0.5 ^o ^C using a re-circulating water bath. At predetermined time intervals, 1ml samples were withdrawn from the receiver compartment and replaced with fresh medium. The amount of imatinib was analyzed by UV spectrophotometry at 260 nm. Each test was performed three times. To evaluate permeability, the mean cumulative amount of imatinib permeated per unit surface area of the rat skin was plotted against time and the permeation flux $\left(\frac{\mathrm{dQ}}{\mathrm{dtA}}\right)$ was calculated using the slope of the linear portion of the plot 

*In-vivo anti-RA activity of imatinib-TFS-Gel*

*Animals*

The 8–10 weeks old male Wistar rats weighing between 180 and 220 g were used in this study. Rats were housed in cages (3 animals per cage) and maintained under standard temperature and light/dark cycle. They were fed with a standard pellet diet and had free access to water. The animal experimental study was approved by the Ethics Committee of Isfahan University of Medical Sciences, Isfahan, Iran.

*RA Induction and monitoring*

Experimental RA was induced as described previously by Nasr *et al.* (13). Briefly, 100 µL of complete Freund’s adjuvant (CFA) containing 10 mg/mL of heat-killed *M. tuberculosis* was injected subcutaneously into the right hind paw of each rat on day 0. After 24 h, animals were divided into four groups (n = 6). Group I received imatinib-Gel (0.1% w/v), group II received imatinib-TFS-Gel (0.1% w/v), Group III and IV received imatinib-free gel and imatinib-free TFS-Gel, respectively. Two hundred milligrams of each formulation were administered topically on the paws of arthritic rats once daily. Just before CFA injection and on days 5, 8, 11 and 14 post-injection, ankle diameters were measured using a digital caliper. The percent increase in ankle diameters at each time used was calculated from the following equation: 

$\mathrm{Percent increase in ankle diameters\%}=(\frac{D_{t}-D_{0}}{D_{0}})\times 100$

Equation 3.

Where D_o_ indicates ankle diameters at day 0 and D_t_ is ankle diameters at any specified time.

On day 14, animals were sacrificed and the left and right hind paws were transected for determination of their weights. The weight differences between the right (RA) and left (contralateral control) paws were used as another index of CFA-induced paw inflammation. 

## Results and Discussion

*Experimentation design*

**Figure 1:**
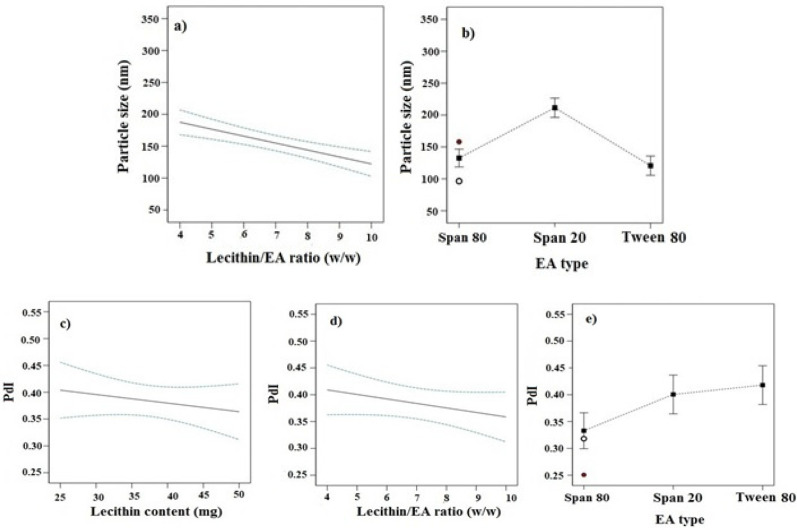
Effects of different studied parameters on PS and PDI of imatinib-TFS

**Figure 2:**
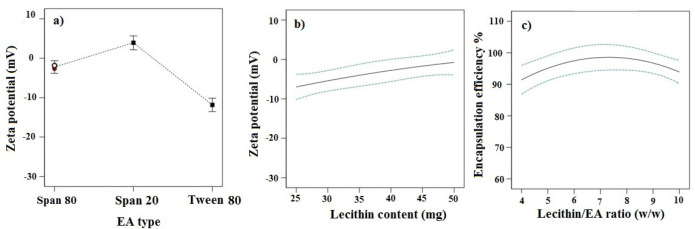
Effects of different studied parameters on ZP and EE % of imatinib-TFS

**Figure 3:**
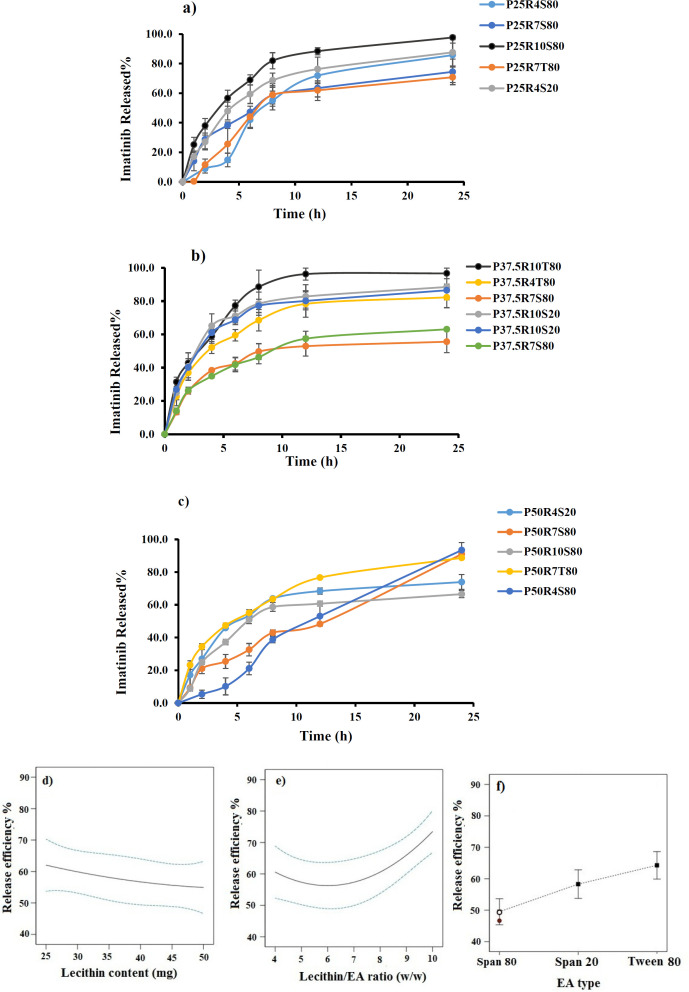
(a-c) Release profile of imatinib from TFS (d-f) Effects of different studied parameters on release efficiency% of imatinib-TFS

In this study, imatinib-TFS were prepared using the film hydration method. The D-optimal design was used to optimize and assess the main effects of formulation variables including lecithin content, lecithin/EA ratio and the type of EA on the PS, PDI, ZP, EE% and RE%, as described in Table 1. The obtained responses from 16 runs, generated by using the Design-Expert software, are shown in Table 2. Analysis of data indicated that the best model fitted to the data obtained for PS and PDI was the 2FI model whereas for ZP, EE and RE, the selected model was quadratic. The lack of fit F-value for these models was not significant, implying that the models were appropriate for prediction within the range of experimental variables. Figures 1-3 indicate the effects of independent variables on studied responses.

*Effect of independent variables on PS and PDI*

As seen in Table 2, the PS varies from 96.4 nm to 334.4 nm. Figure 1 showed the effects of different studied parameters on the PS of imatinib-TFS. Analysis of data indicated that the PS was inversely affected by the lecithin/ EA ratio; as seen increasing lecithin/ EA ratio decreased the PS of imatinib-TFS (*p*-value < 0.05, Figure 1a). This may be attributed to the change in packing density of phospholipid molecules within TFS bilayers caused by the EA (surfactants). Surfactants located in the bilayer may cause incomplete maturation of vesicles and the formation of larger particles. These results are in good agreement with those of Qushawy *et al.* (14) who reported that the PS of miconazole-loaded TFS was reduced when increasing the lecithin/ EA ratio from 80:20 to 90:10. It was found that PS of TFS also was dependent on the molecular structure, HLB, and ionic nature of the surfactant (15). Analysis of our data showed that EA type also had a significant effect on PS (*p*-value < 0.05). As shown in Figure 1b, the PS of TFS comprising different surfactants were in the following order: Tween 80 < Span 80 <Span 20. Compared with span 20 (HLB: 8.6), incorporation of span 80 (HLB: 4.3) considerably decreased the PS. The finding that an EA with a lower HLB value produced vesicles of smaller size may be related to the decrease in the surface energy with the increase in hydrophobicity (16). Despite its higher HLB, Tween 80 (HLB: 15) produced smaller PS than span 80. This can be due to the presence of several ethylene oxide side chains in Tween 80 which provide higher steric repulsion in the continuous aqueous phase, impeding the aggregation of vesicles (16-18). In addition, since the lipophilic part of Tween 80 was shorter than the polyoxyethylene chain as the hydrophilic region, the extent of Tween 80 intercalated into the bilayers was not deep. As a result, a hydrophilic moiety of Tween 80, placed the outer part of the bilayer membrane, increased the TFS particle curvature, while, lipophilic part of that, placed the inner part, did the opposite (17). Thereby, the addition of Tween 80 surfactants overall decreased the PS of TFS. These results were in accordance with the results obtained from studies of Aboud *et al.* (16) and Liu *et al.* (17). 

The PDI is a dimensionless parameter that describes the width of the PS distribution. The PDI is an indicator of particle uniformity and may vary from 0.0 to 1.0. Values close to zero indicate a homogeneous dispersion, and those greater than 0.5 indicate high heterogeneity (19). As represented in Table 2, the PDI values of all formulations were between 0.25 and 0.54. The lipid content, lecithin/ EA ratio and the surfactant type all had significant effects on the PDI of the transfersomal formulations (*p*-value < 0.05). The PDI was reversely dependent on the lecithin content and lecithin/ EA ratios (Figures 1c-1e). As lecithin content and lecithin/ EA ratio increased, the PDI of imatinib-TFS considerably decreased**.** The PDI values of TFS formulations comprising different surfactants were in the following order: Tween 80>span 20>Span 80. Thus, compared with span 20 and Span 80, incorporation of Tween 80 in TFS will increase the PDI of imatinib-TFS. 

*Effects of independent variables on ZP*

ZP refers to total electrical charges that develop at the interface between the dispersed particles and the liquid medium. The magnitude of ZP determines the degree of repulsion between particles of the same charge, and thus the physical stability of a formulation. As indicated in Table 2, the ZP values for different TFS formulations varied from -1.12 to -21.33 mV. The ANOVA analysis demonstrated that EA type and lecithin content had prominent effects on ZP (*p*-value < 0.05). 

TFS consisting of Tween 80 showed the highest absolute value of ZP (Figure 2a). This could be related to their smaller size as discussed earlier. Smaller particles have indeed a greater surface-to-volume ratio, resulting in a higher density of surface charge of the particles.

The increase in lecithin amount led to the decrease in ZP (*p*-value <0.05, Figure 2b). This might be attributed to exposure of the N-terminal sequence of phosphatidylcholine on the outer part of TFS, inducing a higher positive ZP. In this way, increasing lecithin content resulted in an overall decrease of ZPs (20). 

*Effect of independent variables on EE %*

The EE of imatinib formulations ranged from 69.65% to 96.98%. Analysis of data showed that the lecithin/ EA ratio was the only significant factor affecting the EE (*p*-value < 0.05). As shown in Figure 2c, the EE of the drug increased significantly with the increase of lecithin/ EA ratio from 4 to 7. This indicates that higher surfactant amounts, *i.e.* where the lowest lecithin/ EA ratio is used, may decrease the packing density of the bilayer resulting in the lower EE capability of vesicles. However, further increase of lecithin/ EA ratio from 7 to 10, led to the decrease in the EE. The lower EE at a higher ratio of lecithin/ EA ratio may be related to the size of TFS. Since the lipophilic drug are incorporated within the lipid bilayer, the small size of TFS limits space within the bilayer for large amounts of drug to accommodate (21).

*Effect of independent variables on RE%*

The results of imatinib release studies are shown in Figure 3a-3c. The drug release profile from imatinib-TFS showed a biphasic sustained release pattern. The initial faster drug release seems to be related to the imatinib desorbed from the surface of particles, whereas the second phase represents slower diffusion of the drug through the lipid bilayer membrane of TFS. A similar biphasic release pattern was reported for TFS containing tizanidine (22). To ease the comparison of release behavior of different formulations, the RE was calculated and compared (Table 2). The RE values ranged from 50.83 to 83.26.

The ANOVA results indicated that lipid content, surfactant type and lecithin/EA ratio had significant effects on the RE of imatinib-TFS. As shown in Figure 3d-3e, the RE % decreased with increasing lecithin content and increased as the lecithin/ EA ratio increased. A possible explanation for lower drug release at a lower level of lecithin/ EA ratio and a higher level of lecithin may be that the bilayer was more ordered and less leaky, which hindered drug release (23). These results may also be attributed to the PS, where smaller particles provide a larger surface area exposed to the release medium and, decrease the diffusion path length, and thus enhance the drug release rate. This finding was similar to the results of drug release from other nanoparticles (24). The RE% values of imatinib-TFS consisting of different surfactants were in the following order: Tween 80> Span 20 > Span 80 (Figure 3f), which was in agreement with other studies (16, 22). This might be due to the difference in alky chain-length of EAs (surfactants). The EAs of longer chain length showed decreased drug release rate, possibly due to the enhanced molecular ordering of the TFS. In addition, it is assumed that EAs of high hydrophobicity may reduce the probability of formation of transient hydrophilic holes, hence, decrease the RE (23).

*Optimization*

**Figure 4:**
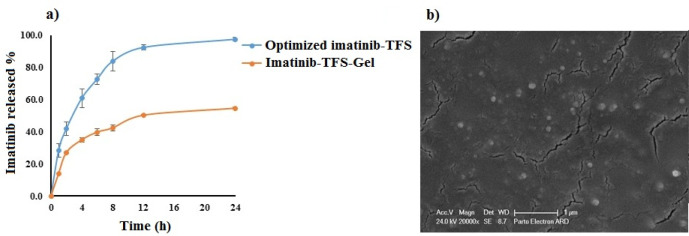
(a) The mean percent of drug release from optimized imatinib-TFS and imatinib-TFS-Gel (b) SEM of optimized imatinib-TFS

The optimum condition for preparation of imatinib-TFS was selected based on the criteria of obtaining minimum values of PS, PDI, and ZP and maximum value of EE and RE. Design-Expert software suggested P_27_R_10_S_80_ as the optimal formulation with a desirability value of 77%. This formulation was composed of 27 mg lecithin, lecithin/ EA ratio of 10 and span 80 as EA. The prepared TFS had PS of 140.53 ± 0.87 nm, PDI value of 0.44 ± 0.01, ZP of -17.63±0.65 mV, EE of 98.70 ± 0.38 % and RE of 81.26 ± 0.70 %. The drug release profile from the optimized imatinib-TFS is shown in Figure 4a. For validation of D-optimal results, the error % was measured after conducting the experiments as given by the software. As shown in Table 3, the experimental observed values of the studied response were in agreement with the predicted value generated by the software, indicating that the optimization technique was trustworthy and rational. The SEM photomicrograph of the imatinib-TFS shows spherical shaped particles with the size in agreement with that obtained by the PS analyzer (Figure 4b)

*FTIR analysis*

**Figure 5:**
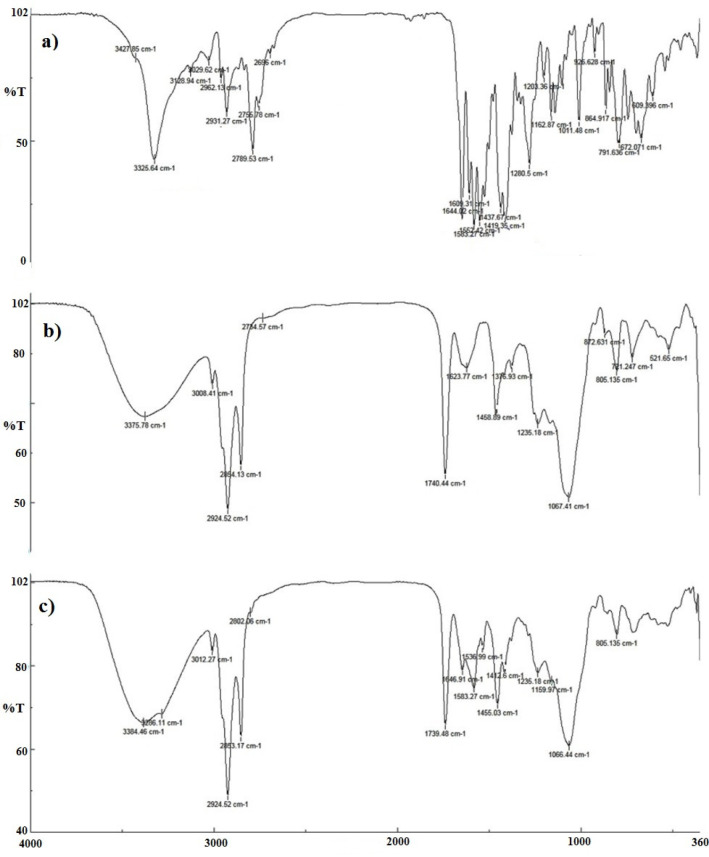
FTIR spectra of imatinib, blank TFS, and imatinib loaded TFS

The FTIR spectra of imatinib, blank TFS, and imatinib-loaded TFS are shown in Fig 5. The pure imatinib shows bands at 3325.64 cm^-1^ (N-H stretch), 2931.27 cm^-1^ (C-H stretch, aromatic), 2789.53 cm^-1 ^(C-H stretch, aliphatic), 1644.02 cm^-1^ (C=O carbonyl), 1583.27 cm^-1 ^(C=C aromatic) and 1552.42 cm^-1^(C=N aromatic). In addition to characteristic bands which were identified in the FTIR spectra of blank TFS, imatinib-TFS showed absorption bands related to imatinib with no significant shift. The results indicated the entrapment of imatinib into the TFS, as well as no physicochemical interaction between imatinib and excipients in TFS formulation.

*Freeze-drying of imatinib-TFS*

The major obstacle limiting the application of nanoparticles is related to their physical and/or chemical instability; this could commonly occur when there is the storage of these nanoparticle aqueous suspensions for a relatively long period. To improve the physical as well as chemical stability of such systems, the removal of water should be done. Freeze-drying is known as the most widely applied process allowing the conversion of solutions or suspensions into solids with adequate stability for distribution and storage in the pharmaceutical field. Freeze-drying is regarded as an industrial process consisting of removing water from a frozen sample through sublimation and desorption in vacuum conditions. In such a process, different stresses may be generated during the freezing and drying stages. Therefore, protectants are usually added to the formulations to prevent or minimize destabilization processes during freezing and desiccation stresses (25). The mean PS of imatinib-TFS which was freeze-dried with and without cryoprotectants after re-dispersion in water can be seen in Table 4. In the formulations lacking cryoprotective agents, a considerable rise in PS and PDI was observed. 

A considerable increase of PS upon reconstitution of freeze-dried TFS sample in all tested samples was observed where mannitol was used as a cryoprotectant in the lyophilization (Table 4). Mannitol is a sugar alcohol with low molecular weight; it can form a crystalline phase through lyophilization. The growing crystals of mannitol could lead to induction of mechanical stress and reduction of the available space for TFS (26-27). TFS which are in such a TFS-rich and poorly hydrated phase may interact more easily, leading to the formation of aggregates. Similarly, Holzer *et al. *(27) indicated that the use of mannitol in freeze-drying increased the mean PS of PLGA nanoparticles following reconstitution, and this was not dependent on the mannitol concentrations applied. Use of 1% w/v lactose and sucrose in lyophilization of TFS produced moderate particle aggregation. The increase of the concentrations of lactose and sucrose to 3% w/v decreased the PS. As shown, formulations with 3% w/v sucrose exhibited almost no change in PS following freeze-drying. Further, it should be noted that imatinib-TFS which were lyophilized with 3% w/v sucrose had the least reconstitution time as compared with other formulations. According to the reconstitution behavior and DLS data following reconstitution, the use of sucrose at 3% w/v seems to efficiently lyoprotect imatinib-TFS during freeze-drying. 

*In-vitro skin permeation investigation*

**Figure 6:**
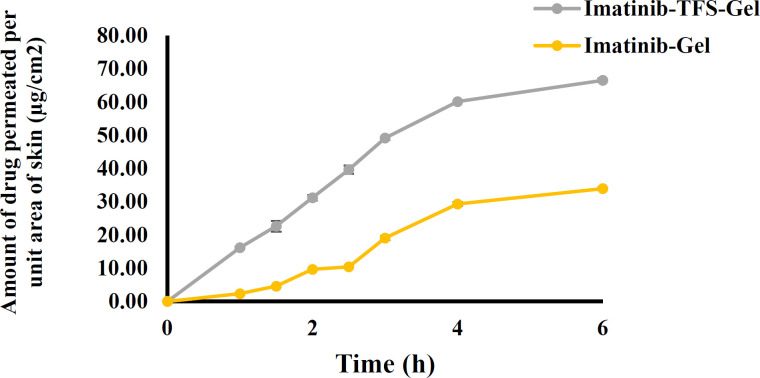
Permeation profiles of imatinib from imatinib-TFS-gel and imatinib-gel through rat skin

The *ex-vivo* skin permeation results are shown in Figure 6. The cumulative amounts of imatinib which permeated through the rat skin from imatinib-TFS-Gel were remarkably higher than from imatinib-gel. The flux of imatinib-TFS-Gel through rat skin was shown to be 15.41 ± 0.12 μg.cm^-2^.h^-1^, whereas imatinib-Gel had a considerably lower transdermal flux (Jss: 7.45 ± 0.20 μg.cm^-2^.h^-1^). The enhanced drug permeation from imatinib-TFS-Gel could be attributed to nanosized TFS. In addition, the presence of the surfactant in the TFS structure may enhance the penetration of vesicles via rendering deformability to TFS (28). TFS can now squeeze themselves through hydrophilic pathways or pores between the skin cells with no loss of the vesicle integrity, (29)

*In-vitro release of imatinib-TFS-Gel*

The release profiles of imatinib from optimized imatinib-TFS and imatinib-TFS-Gel over time are shown in Fig 4a. Imatinib cumulative release during 24 h from TFS-Gel was markedly lower than from TFS (55% *vs.* ~100%). The imatinib slower release from TFS-Gel may be attributed to the obstructive impact of the gel matrix (30-31). Under this condition, the drug was first released from TFS; then its diffusion through the gel matrix occurred; this led to more sustained release as compared to drug release from TFS. This was in agreement with the results obtained by Ali et al (32), who showed that the gel matrix surrounding papaverine hydrochloride-loaded nano-TFS could impede the release of the drug.

*In-vivo* *studies*

**Figure 7:**
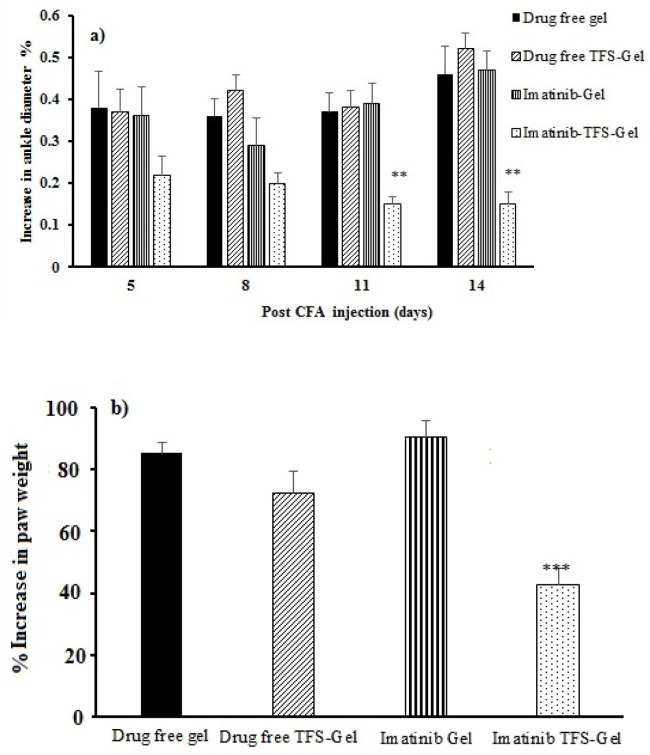
(a) Increase percentage in ankle diameter among diverse treated groups (b) Increase percentage in paw weight among diverse treated groups

The CFA subcutaneous injection produced local edema within a few hours (33). The changes occurred in rat ankle diameter and paw weight were used to evaluate the effects of imatinib on RA. Figure 7a displays the percentage of increase in the ankle diameter among different treatment groups. Swelling in the right paw of the animals which had been induced by CFA injection on the first day was increased until the end of the experiment in drug-free gel, drug-free TFS-Gel, and imatinib-Gel treated groups. The percentage of increase in the rats’ ankle diameter treated with imatinib-TFS-Gel was found to be much less than that with other groups notably on the 11th and 14*th* days of the treatment (*p* < 0.05, Figure 7a). 

Figure 7b shows the percentage of increase in paw weight among different groups on the 14^th^ day of the treatment. The increased percentage in paw weight in the RA animals which were treated with drug-free gel, drug-free TFS-Gel and imatinib-Gel was found to be 85.2%, 75.1% and 90.4%, respectively. The least weight increase was 42.9% which belonged to the animals treated with imatinib-TFS-Gel. This was also in agreement with the results related to ankle diameter, both demonstrating the efficacy of imatinib-TFS-Gel in the treatment of arthritic rats. The more therapeutic efficacy in the developed formula could be attributed to the ability of TFS to increase the imatinib percutaneous permeation. So, a higher amount of the drug could be transferred to deeper skin layers, thus improving the therapeutic anti-inflammatory response. These results were also in agreement with the previous research, where, Lei W *et al.* (34) reported a more efficient treatment of atopic dermatitis where tacrolimus was formulated in TFS.

**Table1:** Independent and dependent parameters employed in D-optimal design for preparation and optimization of imatinib-TFS

**Variables**	**Levels**		
X1 = Lecithin content (mg)	25	37.5	50
X2 = Lecithin/EA ratio (w/w)	4	7	10
X3 = EA Type	Span20	Span80	Tween80
Dependent variables		Constraints	
Particle size (nm)		Minimize	
PDI		Minimize	
Zeta potential (mv)		Minimize	
Encapsulation efficiency%		Minimize	
Release efficiency during 24 h		Minimize	

**Table 2:** Composition of different designed imatinib transfersomal formulations and their observed responses

**Formulations**	**EA type**	**P**	**R**	**Particle size (nm)**	**PDI**	**Zeta potential (mV)**	**Encapsulation efficiency (%)**	**Release efficiency (%)**
P25R4S80	Span 80	25	4	133.91 ± 2.11	0.42 ± 0.02	-3.84 ± 0.13	69.65 ± 0.85	57.82 ± 1.27
P37.5R10T80	Tween 80	37.5	10	127.43 ± 3.46	0.48 ± 0.03	-15.13 ± 0.91	96.03 ± 1.64	83.26 ± 2.14
P50R7S80	Span 80	50	7	146.19 ± 1.64	0.28 ± 0.01	-4.13 ± 0.21	92.16 ± 1.33	50.83 ± 2.25
P50R10S80	Span 80	50	10	151.18 ± 3.71	0.29 ± 0.02	-10.90 ± 0.47	91.79 ± 2.12	53.55 ± 3.42
P50R4S20	Span 20	50	4	334.45 ± 5.96	0.48 ± 0.02	-3.45 ± 0.36	91.12 ± 0.79	59.93 ± 1.09
P37.5R7S80	Span 80	37.5	7	158.14 ± 2.59	0.25 ± 0.01	-2.67 ± 0.15	98.88 ± 0.34	46.65 ± 3.83
P37.5R10S20	Span 20	37.5	10	101.59 ± 1.60	0.29 ± 0.02	-1.82 ± 0.04	82.68 ± 2.80	74.58 ± 3.76
P25R7S80	Span 80	25	7	117.46 ± 2.65	0.42 ± 0.02	-1.12 ± 0.01	90.71 ± 2.74	56.67 ± 3.48
P50R7T80	Tween 80	50	7	99.14 ± 0.98	0.41 ± 0.04	-5.55 ± 0.37	92.82 ± 1.76	67.30 ± 2.65
P37.5R4T80	Tween 80	37.5	4	110.26 ± 1.96	0.41 ± 0.03	-21.33 ± 1.03	96.59 ± 1.00	67.89 ± 2.63
P50R4S80	Span 80	50	4	134.32 ± 2.12	0.27 ± 0.01	-4.53 ± 0.22	75.94 ± 3.10	48.99 ± 2.53
P25R10S80	Span 80	25	10	123.38 ± 2.19	0.41 ± 0.03	-17.41 ± 0.86	96.98 ± 0.20	78.14 ± 1.82
P37.5R10S20	Span 20	37.5	10	105.34 ± 2.53	0.29 ± 0.03	-2.70 ± 0.02	85.59 ± 0.81	72.42 ± 2.46
P37.5R7S80	Span 80	37.5	7	96.46 ± 1.80	0.32 ± 0.04	-1.85 ± 0.06	94.24 ± 2.33	49.31 ± 2.36
P25R7T80	Tween 80	25	7	145.82 ± 2.24	0.36 ± 0.04	-19.20 ± 1.10	93.28 ± 1.66	60.42 ± 2.78
P25R4S20	Span 20	25	4	304.70 ± 4.88	0.54 ± 0.05	-3.96 ± 0.31	89.43 ± 2.45	67.31 ± 4.19

P: Lecithin content (mg); R: Lecithin/EA ratio (w/w); S80: Span 80; T80: Tween 80.

**Table 3:** Comparative levels of predicted and observed responses for the optimized formulation

**Response**	**Particle size (nm)**	PDI	**Zeta potential, (mV)**	**Encapsulation efficiency%**	**Release efficiency%**
Predicted values	120.1	0.386	-15.9	98.8	75.1
Actual values	140.53 ± 0.87	0.44 ± 0.01	-17.63±0.65	98.70 ± 0.38	81.26 ± 0.70
Errors (%)	17.01 ± 0.89	13.03 ± 2.38	-10.90±4.09	-0.10 ± 0.40	8.21 ± 1.14

**Table 4:** Physicochemical properties of imatinib-TFS after freeze-drying with and without cryoprotectants

**Cryoprotectants**	**Concentration%, (w/v)**	**Particle size, (nm)**	**PDI**	**Dispersion time, (s)**
*-*	-	846.13 ± 12.84	0.658 ± 0.019	55.33 ± 2.62
Sucrose	1	235.37 ± 2.9	0.524 ± 0.003	28.33 ± 2.49
	3	173.63 ± 3.4	0.472 ± 0.003	21.00 ± 1.63
Mannitol	1	783.67 ± 5.5	0.286 ± 0.002	60.33 ± 6.60
	3	862.33 ± 72	0.310 ± 0.002	42.67 ± 2.49
Lactose	1	357.97 ± 7.6	0.328 ± 0.003	57.67 ± 6.80
	3	248.50 ± 5.3	0.815 ± 0.044	45.67 ± 4.03

## Conclusion

In the present study, imatinib-TFS was successfully prepared by thin-film hydration method and optimized for size, PDI, ZP, EE% and RE_24 h_% through D-optimal design. The optimal TFS formulation composed of 27 mg lecithin, lecithin/ EA ratio of 10 and span 80 as EA yielded vesicles of least PS, PDI and ZP and highest EE and RE values. The optimized imatinib-TFS formulation incorporated into the Carbopol 940 gel showed enhanced flux of imatinib through the rat skin as compared to imatinib-gel. Animal studies also exhibited improved paw inflammation in the group treated with imatinib-TFS-Gel, as compared to the group treated with imatinib-Gel. Our results demonstrated that transfersomal gel systems provided enhanced drug permeability through rat skin and improved therapeutic response in RA animals, as compared to conventional drug-incorporated gel systems. However, further studies are required to completely evaluate the promising potentials of such delivery systems for topical delivery of drugs, including imatinib in the treatment of inflammatory skin diseases.

## Declaration of conflicting interests

The authors report no conflicts of interest 
